# Working Together to Promote Diabetes Control: A Practical Guide for Diabetes Health Care Providers in Establishing a Working Alliance to Achieve Self-Management Support

**DOI:** 10.1155/2016/2830910

**Published:** 2015-11-22

**Authors:** Allan Jones, Michael Vallis, Debbie Cooke, François Pouwer

**Affiliations:** ^1^Institute of Psychology, University of Southern Denmark, 5230 Odense, Denmark; ^2^CDHA Behaviour Change Institute, Dalhousie University, Halifax, NS, Canada B3H 4R2; ^3^School of Health Sciences, University of Surrey, Guildford GU2 7XH, UK; ^4^Centre of Research on Psychology in Somatic Diseases (CoRPS), Department of Medical & Clinical Psychology, Tilburg University, 5037 AB Tilburg, Netherlands

## Abstract

The quality of the “patient-carer” relationship is the foundation of self-management support and has been shown to influence treatment outcome in relation to psychological and somatic illness, including diabetes. It has long been accepted within applied psychology that the quality of the client-therapist relationship—termed the *working alliance*—is of central importance to treatment outcome and may account for a significant degree of the overall treatment effect. Diabetes healthcare providers have recently expressed a need for further training in communication techniques and in the psychological aspects of diabetes. Could we take a page from the psychological treatment manual on *working alliance* in therapy to guide the diabetes healthcare provider in their role of supporting the person with diabetes achieve and maintain better metabolic control? This paper examines the role of the working alliance in diabetes care and offers a practical guide to the diabetes healthcare provider in establishing a working alliance with the person with diabetes in managing diabetes.

## 1. Introduction

Diabetes mellitus is a widespread chronic disease that has reached epidemic proportions globally [1]. The successful management of diabetes is contingent upon the person with diabetes' ability to achieve glycaemic control through adhering to a demanding daily treatment regimen consisting of taking medication, blood glucose testing, dietary and exercise behaviour, and so forth. Many people with diabetes find it difficult to adhere to the lifestyle- and behavioural- changes necessary to promote effective management of diabetes and are at an increased risk for burdensome complications such as nephropathy, neuropathy, retinopathy, amputation, cardiovascular disease, and other serious conditions.

Many of the behaviours that are required to improve health outcomes are identified by the healthcare provider who recommends and provides education regarding these behaviours to the person in their care. The person receiving care is then faced with the challenge of following-through on the recommendations. In terms of relational dynamics, making recommendations and providing education involve the healthcare provider adopting an expert role, with the person with diabetes typically adopting an uninformed help-seeker role. This relational dynamic is paternalistic in that the healthcare provider is the authority on the person's care and controls the care process, and the role of the person in care is to receive, understand, and follow the direction given. There is evidence to suggest that the quality of the communication between the person with diabetes and the healthcare provider has a strong impact on self-management and clinical outcomes such as A1c. For example, a cross-sectional analysis of almost 10.000 people with diabetes found significant and clinically meaningful relationships between poor communication with healthcare providers and difficulties taking medication, especially oral hypoglycemic medications [2]. Moreover, recent findings from the second Diabetes Attitudes Wishes and Needs (DAWN2) study revealed that only 24% of the people with diabetes surveyed (out of a total of 8596 participants) recalled being asked how diabetes affected their life [3]. The importance of the relationship dynamic between the healthcare provider and the person with diabetes can be appreciated when one considers the issue of motivation and barriers to change. For individuals who present with high motivation to change along with few barriers to change, recommendations and education might be sufficient to result in high adherence to diabetes management behaviours. In cases for which the motivation to change is limited and significant barriers to change are present, why would knowing what to do and how to do it overcome these challenges?

While recommendation- and education-based strategies can be effective in promoting self-management behaviours, the degree of efficacy is often contingent upon third factor variables. For instance, a person's ambivalence to following rigorous treatment recommendations (perhaps due to the burden of maintaining self-care behaviours) as well as the emotional reaction from the appraisal of the meaning and consequences of the recommendations (for example: I do not have the resources to cope with or manage such a change and I am feeling very stressed) can impact on decision-making and self-management [4, 5]. Moreover, there is consistent evidence demonstrating that knowledge alone is insufficient in achieving behaviour change in relation to diabetes self-management [6, 7]. It has also been shown that the interpersonal aspects of communication in diabetes care, such as involvement in decision-making (e.g., the tasks to be performed) and goal setting (e.g., the agreed outcomes), are valued more highly by the person with diabetes than passive acquisition of information [8]. This way of thinking shifts the dynamic of the relationship between the healthcare provider and the person with diabetes from one of paternalism, to one of collaboration, through empowering the person with diabetes to retain their autonomy (sense of control) throughout the care process [5]. Moreover, such participatory approaches have been shown to improve treatment outcomes, such as greater change in personal responsibility for diabetes [9] and improvements in A1c levels and end-organ complications [10]. Communication, therefore, is more than just the transfer of information or skills from one person to another, but a relationship that can impact on how the person with diabetes relates to and engages in their diabetes care.

A person with diabetes' commitment to follow treatment recommendations is partly affected by appraisals regarding the health-care provider's perceived characteristics, such as trustworthiness, integrity, and supportiveness [11]. As such, poor communication may not only produce deficits in knowledge acquisition and consequently the person with diabetes' ability to make informed choices regarding diabetes care, but may also lead the person receiving care to attribute negative characteristics (e.g., unsympathetic) to the caregiver that may obstruct or corrode the “person with diabetes-healthcare provider (PWD-HCP) relationship.” It has long been understood that common factors in psychotherapy (factors not specific to any mode of treatment) such as the client-therapist relationship are of central importance to treatment outcome [12–14]. Common factors may account for up to 45% of the treatment effect across many different psychological conditions [15]. In other words, the specific technique(s) of any psychological intervention only accounts for a portion of the overall treatment effect. Common factors are a ubiquitous part of any interpersonal communication and a component of the professional relationship, whether one is aware of it or not. Psychologists use their understanding of common factors in working with clients to increase the likelihood of helping the client achieve their treatment goals. Focus on common factors, especially the* working alliance*, has become standard practice in applied psychology.

Healthcare providers have recently expressed a need for further training in communication techniques and in the psychological aspects of diabetes [16]. Could we take a page from the psychological treatment manual on common factors in therapy to guide the diabetes healthcare provider in their role of supporting the person with diabetes achieve and maintain better metabolic control? The aim of this paper is to examine the potential role of the working alliance in diabetes care and to offer a practical guide to the diabetes care provider in establishing a PWD-HCP working alliance in managing diabetes.

### 1.1. Working Alliance in Diabetes Care

Common factors in treatment include the person with diabetes' expectations in regard to treatment outcome, beliefs regarding the efficacy of the interventions applied (whether pharmacological, psychological, or other), and the quality of the PWD-HCP relationship, termed the working alliance [15, 17]. When adopting a common factors approach, how the healthcare provider interacts with the person with diabetes is as important as any specific behavioural or biomedical intervention used; that is, the focus is not just on* what* we do but also on* how* we do it. Working alliance is perhaps the best empirically supported common factor in relation to treatment outcome [14, 17–19]. In the context of diabetes management, working alliance can be understood as the collaborative effort between the person with diabetes and the healthcare provider to manage diabetes and prevent further complications, while also trying to reduce the psychological burden that the sustained and arduous management of diabetes can induce. The working alliance between the person with diabetes and the healthcare provider can be divided into three components (based on the working alliance model by Bordin, [20]).

*(1) Tasks*. The cooperative component of the professional relationship encompasses the agreed upon treatment based activities such as measuring blood glucose levels, adjusting insulin doses, taking oral medications, eating more healthily, and maintaining an active lifestyle. Lack of motivation to change often reflects a lack of task alliance between the person with diabetes and the healthcare provider.

*(2) Goals*. The cooperative component of the professional relationship encompasses the agreed upon aims or outcomes of the treatment such as good glycaemic control, lower blood pressure, and low LDL cholesterol, achieving or maintaining a healthier weight. Lack of a goal alliance might be reflected in a case where the person with diabetes wants to live a lifestyle as close to their prediabetes lifestyle as possible (what the person with diabetes describes as being “normal”), while in contrast the healthcare provider wants the person with diabetes to actively engage in diabetes tasks to improve A1c levels. Moreover, lack of goal alliance often results from the healthcare provider focusing near-exclusively on A1c as the main outcome, while the person with diabetes may struggle to understand and make A1c personally relevant, instead focusing on quality of life issues.

*(3) Bond*. The emotional and value-based component of the professional relationship encompass affective appraisals such as trust, warmth, empathy, and acceptance. If the bond alliance is lacking, the person with diabetes might perceive the healthcare provider as judgemental and/or lacking in understanding.

**Figure 1:**
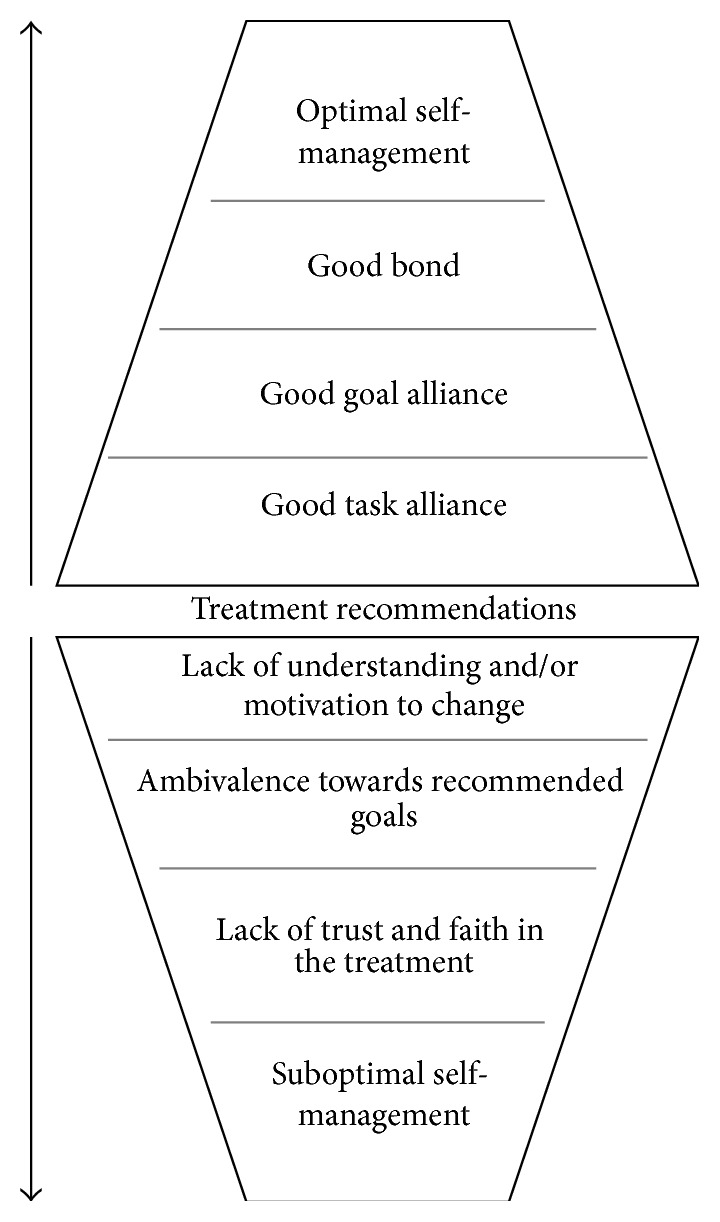
Conceptual model of the role of working alliance in diabetes care.

Although there is strong empirical support for the beneficial effects of the working alliance in the psychological research literature [21], very little is known about the role of working alliance in the context of chronic physical illness, including diabetes care. Working alliance has been found to be significantly associated with more optimal treatment adherence to and greater satisfaction with treatment in a sample of 118 patients diagnosed with a chronic medical illness including diabetes [22] and significantly associated with treatment adherence in people with diabetes [23, 24]. In studies by Attale et al. [25] and Viinamäki et al. [26] working alliance was also found to have a significant positive association with metabolic control in people with type 1 diabetes. Thus, when a good level of collaboration (shared tasks and goals) and a strong bond are established, the active ingredient of the intervention (pharmacological, psychological, or educational) may increase in efficacy through an increase in treatment self-management or through other treatment related factors. In other words, a good working alliance can be understood as a “conditio sine qua non” to effective treatment outcome. Strains or breaks in the working alliance could lead to less optimal self-care behaviours and suboptimal glycaemic control (see Figure 1 for a conceptual model of working alliance in diabetes care). As such, the potential contribution of the working alliance merits serious attention in diabetes care. How then does the healthcare provider establish a good working alliance with the person with diabetes?

### 1.2. Building a Working Alliance

The example below describes how a dialogue between a healthcare provider and a person diagnosed with type 2 diabetes displaying ambivalence in regard to treatment related behaviour change could develop in building a working alliance. 

*HCP:* Perhaps today, in addition to reviewing your glucose levels, we could also focus on other things important to you in regards living with and managing your diabetes. (The HCP modifies the usual treatment agenda to negotiate a collaborative relationship. Note: prioritising the rigid application of the technique or treatment protocol over the PWD's unique needs is an incomplete care approach, which can result in a strained alliance and poor treatment outcome [12].)

*PWD*: That would be great. I know I'm not the best patient… I cannot seem to stick to the recommended diet.

*HCP*: Would you consider being less hard on yourself? It's not easy to change habits, and I am here to help (here the HCP is communicating an understanding of the patient's situation and signalling that “we” (see Frishman [27]) are working together towards achieving the treatment goals [Bond]).

*PWD*: I just cannot seem to stay away from junk-food, even though I know it's bad for me…and if I continue down this path I know things are only going to get worse.

*HCP*: You are worried about things getting worse. Are there things we could change to avoid things from getting worse? (The* task* is to regulate diet, the* goal* is to stop “things getting worse”).

Working alliance in the medical setting can be measured using a reworded short client version (to reflect the medical relationship—see Fuertes et al. [22]) of the Working Alliance Inventory (WAI-12: [28, 29]). The WAI contains 12 items, measured on a 7-point scale, and includes three subscales:* tasks*, items that measure the degree to which patient and provider agree on the actions to be carried out in the treatment process;* goals*, items that measure the degree to which patient and provider agree on what is to be achieved through following the treatment regimen; and* bond*, items that measure the degree of trust, acceptance, and belief in the healthcare provider's recommendations. The WAI-12 has been found to have acceptable psychometric properties when used as an overall (single factor) measure of the patient-provider working alliance [22].

*Empathy* towards the person with diabetes is arguably one of the most important evidence-based factors in the PWD-HCP relationship [30–32]. Empathy is similar in many respects to other interpersonal constructs such as warmth and acceptance and has a supportive function. However, empathy also involves the ability and the willingness of the healthcare provider to understand the person with diabetes' unique situation, to identify (*Ask*) and understand (*Listen*) how the person with diabetes sees and feels things [5] and to communicate this understanding when interacting with the person with diabetes (*Summarise*) [30–33]. The ability of the healthcare provider to communicate empathy is not only linked to the onset and maintenance of the PWD-HCP bond (the affective component of the relationship) but also contributes to establishing consensus on the tasks and goals to be included in the treatment process through a communicated understanding of the person with diabetes' unique situation [30, 31]. Empathy, as expressed using the* Ask*,* Listen*, and* Summarise* approach, then empowers the healthcare provider to* Invite* the person with diabetes to consider new information, such as specific diabetes management strategies.* Ask*,* Listen*,* Summarise*, and* Invite* is a relational dynamic that takes advantage of the principle of relational complementarity [34, 35], referring to the circumplex model of relationship functioning, in which affiliative behaviours are likely to be reciprocated. In other words, the best way to encourage someone to listen to you is to first listen to them.

Reach [5] describes empathy in this context as helping the person with diabetes to elucidate their preferences in relation to the treatment process. Although there are numerous studies showing a positive association between therapist-empathy and good treatment outcome in the psychotherapy literature [30, 31], studies examining this association in relation to diabetes care are scarce. However, a study by Hojat et al. [36] found that healthcare providers measuring high on levels of empathy had a significantly higher proportion of individuals in their care displaying good control of A1c (16%) and low-density lipoprotein cholesterol (15%) compared to healthcare providers with low empathy scores. Similar findings were also reported in a study by del Canale et al. [37], with significantly lower rates of metabolic complications (hyperosmolar state, diabetic ketoacidosis, and diabetic coma) found in patients of physicians measuring high in empathy compared to patients of physicians with moderate and low empathy. Communicating empathy is therefore an important component in diabetes care. The example below describes how a dialogue between a healthcare provider and a person with type 1 diabetes who has a fear of hypoglycaemia could develop in communicating empathy.

### 1.3. Expressing Empathy through Verbal Communication

*PWD*: I'm really worried that if I stay within the range you recommend that I may go low… and then I do not know what might happen.

*HCP*: Sounds like you are feeling anxious about taking your insulin as recommended, as this may lead to a severe hypo.

*PWD*: Yes… or worse. I have children and a husband. I've called in sick to work twice already this year. I'm worried about getting fired, and my family depend on me.

*HCP*: You are afraid something negative might happen to you, and that your family will be left to fend for themselves. I can see how that makes you reluctant to increase your dose of insulin.

*PWD*: Yes (tearfully).

Elliott et al. [31] have come up with recommendations on how to build a client-therapist relationship based on empathy. Based on these recommendations the following points of guidance in building a working alliance with the person with diabetes are offered. (1) Try to step into the person with diabetes' shoes and to understand the* how* and the* why* of the person's experiences, as well as communicating this understanding back to the person with diabetes (see the above example). (2) Showing an understanding of events from the person with diabetes' viewpoint does not mean simply repeating or reframing what the person says but trying genuinely to understand the individuals' perspective, motivations, and concerns in the moment. (3) Communication is both verbal and nonverbal, and coldness or warmth can be easily communicated both verbally and nonverbally. A genuine interest in the person with diabetes' psychological wellbeing and in understanding their experiences can be perceived by the person, even if not yet expressed verbally. (4) Do not easily assume that you correctly understand the person with diabetes' views or that they share your views. Communicating with a certain degree of uncertainty allows the person with diabetes to provide corrective feedback (e.g., well…not really…it's more like…). Also, do not assume that the person with diabetes understands you, even if they appear to understand. Finally, do not assume that because you understand the person with diabetes that the person feels understood.

Empathy in the medical setting can be measured using the Jefferson Scale of Empathy—Health Professional (JSE-HP [38]). The JSE-HP contains 20 items, measured on a 7-point Likert scale, with higher scores indicating the ability of the healthcare provider to communicate in an empathic way when interacting with the person in their care. For example, “*Health care providers should try to stand in their patients' shoes when providing care to them.*” The JSE has been found to have acceptable psychometric properties in measuring empathy in healthcare providers [38–41].

Potential barriers to healthcare providers focusing on developing* bond*,* task*, and* goal* alliance are that it is time-consuming and might elicit distress in the healthcare provider when they begin to understand the intensity of the burden that diabetes might convey on a person. While these barriers are understandable in a busy biomedically oriented practice, our experience in training healthcare providers in these methods is that these barriers resolve themselves over time. That is, after the 10th or 20th person with diabetes in which these methods are used, most healthcare providers start to value the time spent on forming a working alliance and see it as time well invested. Once a person's story is understood, that story tends to carry itself forward in time (i.e., the story only has to be told once) and the agreed upon tasks and goals based on this understanding tend to be more realistic for the person with diabetes.

## 2. Summary

The aim of this paper is to empower the diabetes healthcare provider to better support individuals with diabetes in managing their blood glucose levels through understanding the importance of the working alliance in diabetes care. There are always limitations to any treatment approach and some people with diabetes will continue to experience difficulty in managing their diabetes irrespective of the strength of the working alliance or the therapeutic approach used. By adopting a common factors approach, the healthcare provider will be better equipped to support the person with diabetes in living with and managing their diabetes. Communication based on empathy is likely to act as a catalyst for improved treatment self-management and the adoption of behaviors that facilitate change and lead to increased wellbeing in the person with diabetes. Corrosion in the working alliance and/or low empathy in the communication between the healthcare provider and the person in their care may lead to increased risk of disengagement from treatment and poorer metabolic control for the person with diabetes.
